# The Incremental Value of Copeptin for the Early Rule-Out of Non-ST Elevation Myocardial Infarction in the Emergency Department

**DOI:** 10.3390/jcm15093251

**Published:** 2026-04-24

**Authors:** Sofia Bezati, Christos Verras, Vasiliki Bistola, Dionysis Matsiras, Estela Kiouri, Lambros Markos, Ioannis Ventoulis, Effie Polyzogopoulou, John Parissis

**Affiliations:** 1 Department of Emergency Medicine, Attikon University Hospital, Rimini 1, 12462 Athens, Greece; sofiabezati@gmail.com (S.B.); christos.verras@gmail.com (C.V.); vasobistola@yahoo.com (V.B.); mats.dionysis@gmail.com (D.M.); estelakiouri@hotmail.com (E.K.); lambrosmrk@gmail.com (L.M.); effiep@live.com (E.P.); 2 Department of Occupational Therapy, University of Western Macedonia, Keptse Area, 50200 Ptolemaida, Greece; iventoulis@uowm.gr

**Keywords:** copeptin, high-sensitivity cardiac troponin T, dual marker strategy, non-ST elevation myocardial infarction, rule-out

## Abstract

**Background/Objectives:** Copeptin, a marker of endogenous stress, has been used for the early detection of non-ST elevation myocardial infarction (NSTEMI) in combination with conventional cardiac troponin. However, its incremental diagnostic value, when combined with high-sensitivity troponin, is not well defined. This study seeks to assess the diagnostic performance for NSTEMI of a Dual Marker Strategy (DMS) [copeptin and high-sensitivity cardiac troponin T (hs-cTnT)] measured upon presentation to the Emergency Department (ED) and compare it to the hs-cTnT 0h/1h and 0h/2h algorithms recommended by the European Society of Cardiology (ESC). **Methods:** This prospective observational study enrolled 102 patients presenting to the ED with chest pain of <6 h duration; patients with ST elevation myocardial infarction (STEMI) were excluded. Copeptin and hs-cTnT were measured upon patient presentation (time 0 h, DMS) in the whole cohort. hs-cTnT was subsequently repeated either at 1 h (n = 51) or 2 h (n = 51). The diagnostic performance of the DMS, assessed in terms of sensitivity, specificity, and negative (NPV) and positive predictive value (PPV), was compared to that of the ESC-recommended hs-cTnT algorithms 0h/1h and 0h/2h for NSTEMI. **Results**: Of the total population, 59.8% were men, with a mean age of 57.7 ± 18.4 years; 8.8% of the patients were eventually di agnosed with NSTEMI. The DMS (cut-offs: copeptin < 10 pmol/L and hs-cTnT < 14 ng/L) demonstrated a sensitivity of 88.9% (95% CI: 51.75–99.72) and an NPV of 98.5% (90.94–99.76). On the other hand, the hs-cTnT 0h/1h algorithm showed a sensitivity of 60% (14.66–94.73) and an NPV of 95.6% (88.06–98.45), while the hs-cTnT 0h/2h algorithm exhibited a sensitivity of 75% (19.41–99.37) and an NPV of 95.8% (85.22–98.93). In ROC analysis, copeptin yielded an AUC of 0.702 (*p* = 0.046) and hs-cTnT at 0h showed an AUC of 0.736 (*p* = 0.02), whereas their combination demonstrated an AUC of 0.730 (*p* = 0.023) for the detection of NSTEMI. **Conclusions:** The copeptin/hs-cTnT DMS has comparable diagnostic performance to the hs-cTnT 0h/1h and 0h/2h algorithms for the early rule-out of NSTEMI.

## 1. Introduction

Early identification of patients with Acute Coronary Syndrome (ACS) in the Emergency Department (ED) is a major clinical priority. Among patients with chest pain (accounting for approximately 5% of ED visits [1]), only a small proportion are ultimately diagnosed with ACS, while nearly half are discharged with minor diagnoses [2,3,4]. Given the considerable workload of the EDs and the time-dependent clinical deterioration of patients with ACS, which adversely affects morbidity and mortality, early risk stratification of patients presenting with undifferentiated chest pain cannot be overemphasized. Whereas the diagnosis of ST elevation myocardial infarction (STEMI) is typically straightforward, the diagnosis of unstable angina (UA) and non-ST elevation myocardial infarction (NSTEMI) remains more challenging, often necessitating the use of additional diagnostic tools, including the application of cardiac troponin-based algorithms [3,4].

Due to atypical clinical presentations, patients with NSTEMI often encounter significant diagnostic delays, resulting in extended length of stay in the ED [5,6,7] and delayed delivery of recommended treatment [8,9]. Accelerated rule-in and rule-out biomarker strategies based on high-sensitivity cardiac troponins (hs-cTn) have been introduced to overcome these challenges in the management of NSTEMI. However, such strategies require serial hs-cTn measurements, thereby precluding the diagnosis of NSTEMI immediately upon initial patient presentation. Accordingly, ongoing research has focused on identifying and incorporating novel biomarkers into the initial diagnostic workup of patients with suspected NSTEMI, in combination with hs-cTn. By using a Dual Marker Strategy (DMS), the aim is to reduce the time from first medical contact to NSTEMI diagnosis or rule-out.

Copeptin is a peptide generated upon cleavage of the prohormone of arginine vasopressin (proAVP) and is released in equimolar amounts with arginine vasopressin (AVP) in response to hemodynamic and osmotic changes or stressful stimuli. While its physiological role remains unclear, accumulating evidence suggests that elevated copeptin levels reflect endogenous stress in acute illness and cardiovascular disease [10]. Therefore, the 2015 guidelines of the European Society of Cardiology (ESC) for the management of ACS in patients presenting without persistent ST-segment elevation have recommended its measurement in conjunction with conventional cardiac troponin as part of the initial diagnostic workup of patients with symptoms suggestive of acute myocardial infarction (AMI) [11,12,13]. However, the introduction of accelerated diagnostic protocols for NSTEMI using high-sensitivity troponins (0h/1h and 0h/2h ESC algorithms) has mitigated initial enthusiasm regarding the diagnostic utility of combined measurement of copeptin.

The aim of the present study was to examine the diagnostic performance of a DMS combining copeptin with high-sensitivity cardiac troponin T (hs-cTnT), measured upon initial presentation of patients with chest pain to the ED, for the early rule-out and rule-in of NSTEMI, and to compare its performance with the established hs-TnT 0h/1h and 0h/2h algorithms.

## 2. Materials and Methods

### 2.1. Study Design and Population

This was a prospective, randomized diagnostic accuracy pilot study conducted in the ED of Attikon University Hospital, Athens, Greece, from September 2021 to October 2023. We enrolled 102 patients aged over 18 years, presenting with symptoms suggestive of ACS, such as chest pain with a duration of less than 6 h or acute chest discomfort accompanied by dyspnea, diaphoresis and pain radiating to the left and/or right arm. Exclusion criteria were persistent ST-segment elevation on the electrocardiogram (ECG) and history of end-stage renal disease. The study protocol was developed in accordance with the Standards for Reporting Diagnostic Accuracy (STARD) guidelines for diagnostic accuracy studies [14] and complied with the principles of the Declaration of Helsinki. Ethical approval was granted by the Institutional Review Board of Attikon University Hospital (IRB 347, 2 July 2021). Upon confirmation of eligibility criteria, all patients provided written informed consent prior to study inclusion.

### 2.2. Study Procedures

Upon presentation, information regarding chest pain characteristics, including onset and duration, as well as past medical history, was collected. Clinical assessment included vital signs, physical examination, 12-lead ECG, echocardiography, chest radiography and venous blood sampling for routine laboratory analyses and measurement of cardiac biomarkers, including high-sensitivity cardiac troponin T (hs-cTnT) and copeptin.

hs-cTnT was measured upon patient presentation in all patients (time_0h, hs-cTnT_0h). Following initial assessment, patients were randomized in two groups and were evaluated for the presence of Acute Coronary Syndrome (ACS), according to the European Society of Cardiology (ESC) diagnostic algorithms 0h/1h and 0h/2h [15]. The first group (group 1, n = 51) was assessed according to the 0h/1h algorithm; thus, a second measurement of hs-cTnT was performed 1h after presentation (0h/1h algorithm). The second group (group 2, n = 51) was assessed according to the 0h/2h algorithm; thus, a second measurement of hs-cTnT was done 2h after presentation (0h/2h algorithm). For group allocation, we used a simple random sampling method, by using sealed envelopes, so as to ensure equal probability of assigning a patient to either the 0h/1h or 0h/2h algorithm.

Copeptin was measured only at a single time point upon ED presentation (time_0h) using a subsample of the venous blood drawn at presentation (cpn_0h). The subsample was centrifuged and the serum was stored at −80 °C for subsequent copeptin analysis.

**Figure 1:**
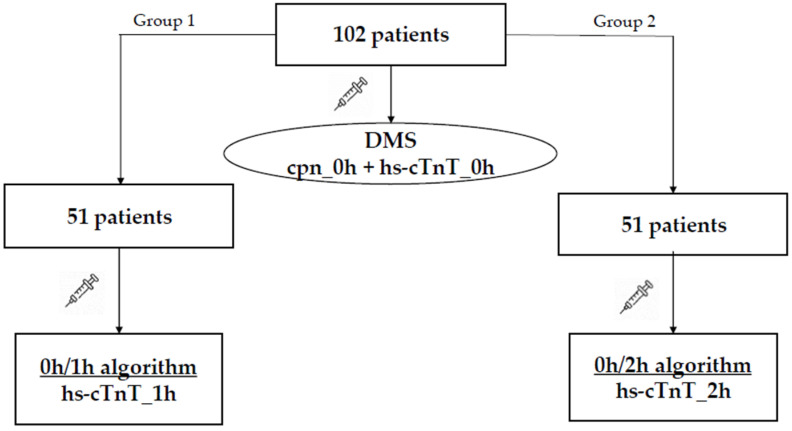
Flowchart illustrating the study protocol. cpn_0h = copeptin upon presentation (time 0 h); DMS = Dual Marker Strategy; hs-cTnT_0h = high-sensitivity cardiac troponin T upon presentation (time 0 h); hs-cTnT_1h = high-sensitivity cardiac troponin T measured 1h after presentation; hs-cTnT_2h = high-sensitivity cardiac troponin T measured 2h after presentation.

The study protocol is illustrated in Figure 1.

The initial diagnosis of ACS in the ED was made by the consultant cardiologist based on clinical presentation, ECG findings and ESC hs-cTnT algorithms. The final diagnosis of ACS was adjudicated by two independent cardiologists after comprehensively reviewing all available data collected during the index hospitalization, including the results of the coronary angiography. Copeptin measurements were not used to guide patient management.

### 2.3. Definitions and Diagnosis of Acute Coronary Syndromes

ACS diagnosis was based on the definitions and criteria outlined in the 2020 ESC guidelines for the management of ACS, with patients being further stratified according to ECG findings into two categories, namely STEMI and NSTEMI [15]. UA was diagnosed when patients exhibited symptoms and/or ECG findings suggestive of myocardial ischemia in the presence of normal levels of cardiac troponin and a culprit lesion on coronary angiography. AMI was diagnosed according to the Fourth Universal Definition of Myocardial Infarction as *“a rise and/fall of cardiac troponin with at least one value above the 99th percentile upper reference limit (URL) and with at least one of the following: a. Symptoms of acute myocardial ischaemia; b. New ischaemic ECG changes; c. Development of pathological Q waves; d. Imaging evidence of new loss of viable myocardium or new regional wall motion abnormality in a pattern consistent with an ischaemic aetiology; e. Identification of a coronary thrombus by angiography including intracoronary imaging or by autopsy”* [16].

To evaluate troponin kinetics for the diagnosis of AMI, the ESC algorithms 0h/1h and 0h/2h were applied. According to the 0h/1h algorithm, AMI was ruled out in patients with very low levels of hs-cTnT upon presentation (hs-cTnT < 5 ng/L) or in those with low baseline levels of hs-cTnT and no considerable change (Δ) at one hour (hs-cTnT < 12 ng/L and 1hΔ < 3). Conversely, AMI was ruled in when patients demonstrated high levels of hs-cTnT upon presentation (hs-cTnT ≥ 52 ng/L) or when they exhibited a considerable change in hs-cTnT levels at one hour (1hΔ ≥ 5).

According to the 0h/2h algorithm, patients were ruled out in case of very low levels of hs-cTnT at presentation (hs-cTnT < 5 ng/L) or in the event of low baseline levels of hs-cTnT and no considerable change (Δ) at two hours (hs-cTnT < 14 ng/L and 2hΔ < 4). On the other hand, patients were ruled in if hs-cTnT levels upon presentation were high (hs-cTnT ≥ 52 ng/L) or if there was a considerable change in hs-cTnT levels at two hours (2hΔ ≥ 10).

It needs to be noted that early rule-out based on a single very low hs-cTnT value was deemed feasible only on the occasion that patients presented to the ED more than 3 h after chest pain onset, in which case either algorithm could be applied [15].

**Figure 2:**
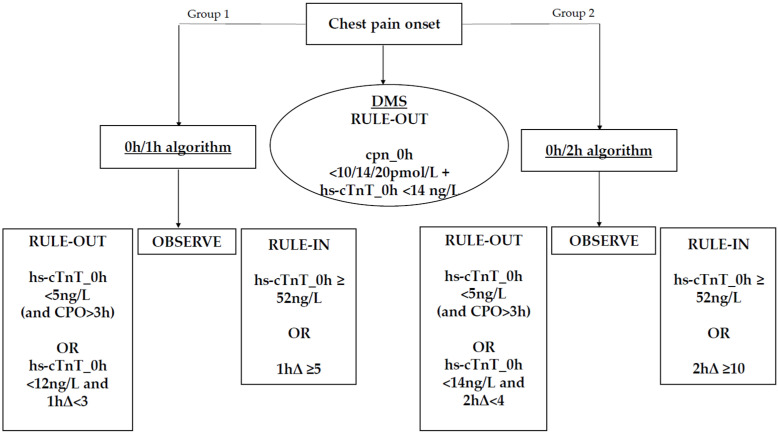
Flowchart illustrating the diagnostic algorithms 0h/1h, 0h/2h and the Dual Marker Strategy (DMS). cpn_0h = copeptin upon presentation (time 0 h); CPO = chest pain onset; DMS = Dual Marker Strategy; hs-cTnT_0h = high-sensitivity cardiac troponin T upon presentation (time 0 h); hs-cTnT_1h = high-sensitivity cardiac troponin T measured 1h after presentation; 1hΔ = absolute change in hs-cTnT levels at 1h; 2hΔ = absolute change in hs-cTnT levels at 2h.

A schematic overview of the diagnostic pathways is provided in Figure 2.

### 2.4. Measurement of Cardiac Biomarkers

hs-cTnT was measured in fresh ethylenediaminetetraacetic acid (EDTA) serum samples using a COBAS 8000 analyzer (Roche Diagnostics GmbH, Mannheim, Germany). The limit of blank (LoB), limit of detection (LoD) and limit of quantification (LoQ) were 3 ng/L, 5 ng/L and 13 ng/L, respectively [17]. The 99th percentile URL in a healthy population is 14 ng/L and was applied as the diagnostic cut-off [18].

Copeptin levels were determined from frozen EDTA serum samples. Measurements were performed on a BRAHMS KRYPTOR COMPACT PLUS analyzer (ThermoFisher Scientific, Waltham, MA, USA) by means of a Time-Resolved Amplified Cryptate Emission (TRACE) immunofluorescence assay. As per the manufacturer’s specifications, the lower limit of detection is 0.88 pmol/L, the functional assay sensitivity is <1.08 pmol/L and the measurement range (with automatic dilution) is 2.7–2.000 pmol/L. Results are available in less than 15 min [19]. For ruling out NSTEMI with the use of the DMS, levels of both copeptin and hs-cTnT were required to be below predefined pathological thresholds. For copeptin, 3 different cut-off values were tested based on the previously published literature [12,20,21]: <10 pmol/L, <14 pmol/L and <20 pmol/L. These thresholds were selected because they have been found to correspond to the 95th, 97.5th and 99th percentile of a healthy population, according to the Gutenberg Heart study [20].

### 2.5. Statistical Analysis

Categorical variables are presented as frequencies and percentages. Continuous variables are reported as mean ± standard deviation (SD) for normally distributed variables, and as median with interquartile range (IQR) for non-normally distributed parameters. Normality was assessed by means of the Kolmogorov–Smirnov test. Comparisons between groups were performed using the Mann–Whitney U test for continuous variables and the Chi-square test for categorical variables, as appropriate. The diagnostic performance of each strategy for the evaluation of ACS was assessed by calculating sensitivity, NPV, specificity and PPV, based on 2 × 2 contingency tables. The values of the corresponding metrics are reported with 95% confidence intervals (CIs). The overall diagnostic performance of copeptin and hs-cTnT was determined by using Receiver Operating Characteristic (ROC) curve analysis, expressed as the Area Under the Curve (AUCs). Logistic regression analysis was performed to evaluate the effect of each variable on the diagnosis of ACS, while ROC curve analysis was employed in order to estimate their combined diagnostic performance. For comparison of ROC curves, the DeLong test was performed. SPSS version 25.0 (IBM Inc., Chicago, IL, USA) and MedCalc Software version 23.4.8 were used for statistical analysis. A *p*-value of <0.05 was regarded as statistically significant.

## 3. Results

A total of 102 patients were included in the study. The mean age was 57.7 ± 18.4 years, and 59.8% of the population were male. Twenty-five patients (24.5% of the total population) presented within 3 h of symptom onset. Demographics, past medical history including comorbidities and concomitant medication, baseline clinical, laboratory and ECG characteristics, as well as chest pain features upon presentation are summarized in Table 1.

NSTEMI was the final diagnosis in nine patients (8.8%). Other causes of chest pain included stable coronary artery disease (5.9%), other cardiac conditions (7.8%) and non-cardiac diseases (77.5%). Patients with NSTEMI had significantly higher levels of hs-cTnT and copeptin at presentation compared to patients who presented with other etiologies of chest pain [hs-cTnT, median (IQR): 32.3 (7–82) ng/L vs. 6.30 (4–11.1) ng/L, *p* = 0.020; copeptin: 18.11 (14.6–22.4) pmol/L vs. 6.99 (4.5–10.1) pmol/L, *p* = 0.046, respectively]. Moreover, patients with NSTEMI had significantly higher HEART scores compared to those without NSTEMI [7 (6–7) vs. 3 (2–4), *p* < 0.001, respectively] and presented earlier to the ED, with seven out of nine patients (77.8%) seeking medical care in <3 h from chest pain onset compared to 18 out of 93 patients (19.3%) without NSTEMI (*p* < 0.001). Adverse events during follow-up included one death in group 1 (i.e., patient group allocated to the 0h/1h algorithm), which was attributed to sepsis.

### 3.1. Diagnostic Performance of hs-cTnT and Copeptin as Single Biomarkers and of the DMS (hs-cTnT Plus Copeptin) upon Presentation

In the total population, hs-cTnT at a cut-off of 14 ng/L upon presentation yielded a sensitivity of 66.7% (95% CI 29.9–92.5), an NPV of 96.3% (95% CI 91.2–98.5), a specificity of 83.9% (95% CI 74.8–90.7) and a PPV of 28.5% (95% CI 17.2–43.4) (Table 2).

Copeptin as a single biomarker demonstrated a sensitivity of 33.4–77.8% and an NPV of 93.2–97.5%, depending on the cut-off used. The highest diagnostic accuracy (83.3%) was observed when 14 pmol/L was used as a cut-off; sensitivity was 77.8% (95% CI 40–97.2), NPV was 97.5 (95% CI 92–99.2), specificity was 83.9 (95% CI 74.8–90.7) and PPV was 31.7 (95% CI 20.7–45.4).

On the other hand, the DMS upon presentation yielded a sensitivity of 66.7–88.9% and an NPV of 96–98.6%, depending on the cut-off of copeptin applied. The highest diagnostic accuracy was observed at a copeptin cut-off of 14 pmol/L. The application of the DMS, when using a copeptin cut-off of <14 pmol/L in combination with a hs-cTnT cut-off <14 ng/L, yielded a sensitivity of 88.9% (95% CI 51.7–99.7) and an NPV of 98.6% (95% CI: 91.8–99.8) for the detection of NSTEMI. Comparable results were observed when using a copeptin cut-off of <10 pmol/L, which, when combined with hs-cTnT < 14 ng/L, demonstrated a sensitivity of 88.9% (95% CI 51.7–99.7) and an NPV of 98.5% (95% CI:90.9–99.8).

In group 1 (i.e., patients allocated to the 0h/1h algorithm), the DMS based on a copeptin cut-off of <14 pmol/L demonstrated better sensitivity (100% [95% CI: 47.8–100]) and NPV (100% [95% CI: 89.1–100]) compared to the hs-cTnT 0h/1h algorithm (sensitivity 60% [95% CI: 14.7–94.7] and NPV 95.6% [95% CI: 88.1–98.5]) for the diagnosis of NSTEMI (Table 3).

**Figure 3:**
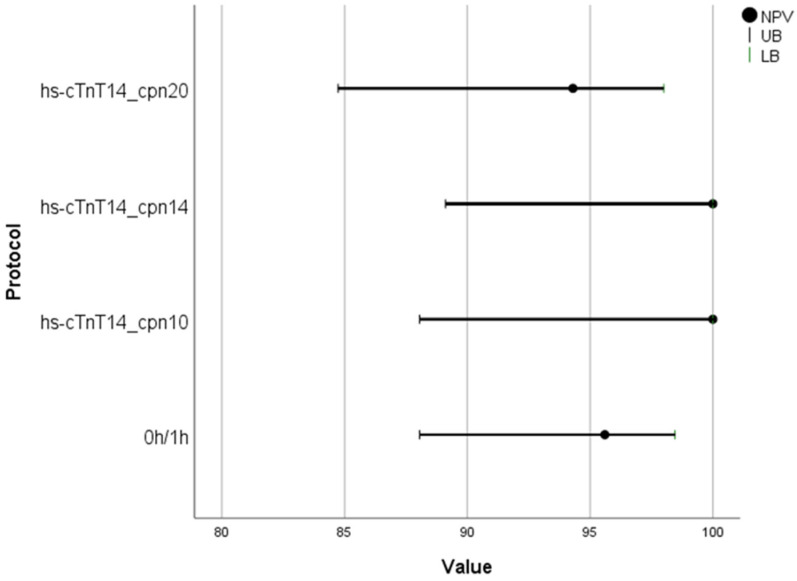
Negative predictive values (NPVs) with corresponding 95% confidence intervals of the Dual Marker Strategy (DMS) across the different copeptin cut-offs applied (cpn < 10 pmol/L, <14 pmol/L or <20 pmol/L) in combination with high-sensitivity cardiac troponin T (hs-cTnT) < 14 ng/L in patients allocated to the 0h/1h algorithm (group 1). Results are presented alongside those of the hs-cTnT 0h/1h algorithm for comparison. LB = lower bound; UB = upper bound.

Figure 3 illustrates the NPVs of the DMS across the different copeptin cut-offs applied in comparison to the hs-cTnT 0h/1h algorithm in group 1.

In group 2 (i.e., patients allocated to the 0h/2h algorithm), the DMS demonstrated similar, yet slightly enhanced, diagnostic performance compared to the hs-cTnT 0h/2h algorithm. While sensitivity was identical for both algorithms (75% [95% CI: 19.4–99.4]), the NPV of the DMS ranged from 97.2% to 97.6% across the three copeptin cut-offs tested and was superior to the NPV of the hs-cTnT 0h/2h algorithm (95.8% [95%CI: 85.2–98.3]) (Table 4).

**Figure 4:**
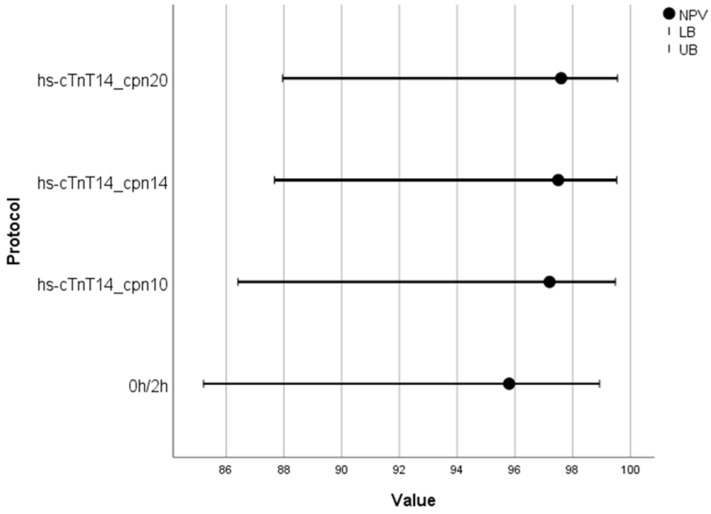
Negative predictive values (NPVs) with corresponding 95% confidence intervals of the Dual Marker Strategy (DMS) across the different copeptin cut-offs applied (cpn < 10 pmol/L, <14 pmol/L or <20 pmol/L) in combination with high-sensitivity cardiac troponin T (hs-cTnT) < 14 ng/L in patients allocated to the 0h/2h algorithm (group 2). Results are presented alongside those of the hs-cTnT 0h/2h algorithm for comparison. LB = lower bound; UB = upper bound.

Figure 4 shows the NPVs of the DMS across the different copeptin cut-offs applied, in comparison to the hs-cTnT 0h/2h algorithm in group 2.

### 3.2. ROC Analysis

**Figure 5:**
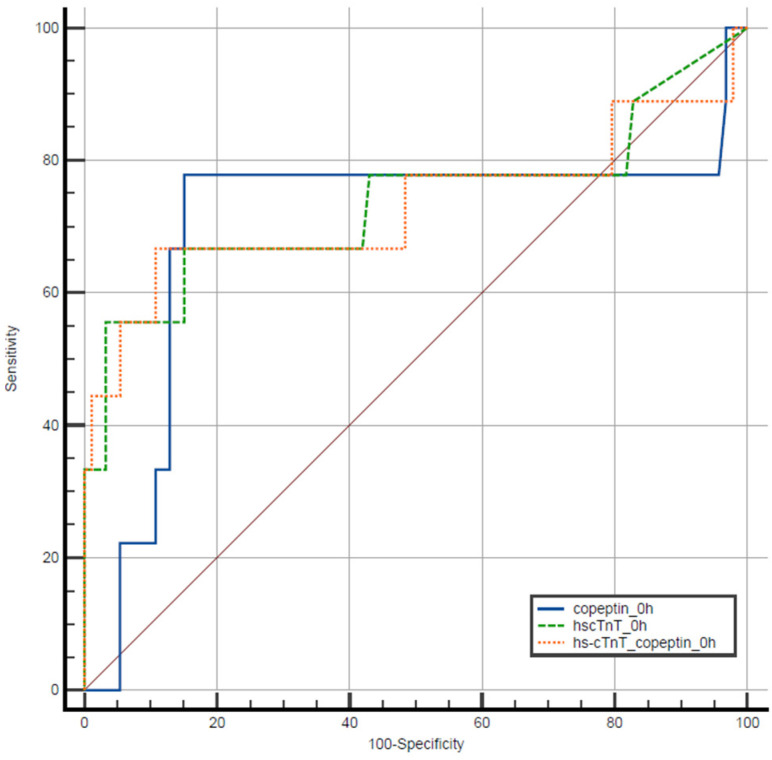
Receiver Operating Characteristic (ROC) curves of copeptin, high-sensitivity cardiac troponin (hs-cTnT) and the combined measurement of copeptin and T upon patient presentation for the diagnosis of non-ST elevation myocardial infarction (NSTEMI).

Using ROC analysis, the overall diagnostic performance of copeptin measured upon presentation (copeptin_0h), expressed as AUC, was 0.702 (95% CI: 0.463–0.94; *p* = 0.046). The AUC for hs-cTnT measured upon presentation (hs-cTnT_0h) was 0.736 (95% CI: 0.506–0.966; *p* = 0.02). The combination of copeptin_0h and hs-cTnT_0h yielded an AUC of 0.730 (95% CI: 0.491–0.969; *p* = 0.023). A comparison of the three ROC curves using the DeLong test is illustrated in Figure 5. The discriminatory ability of copeptin, hs-cTnT or their combination was comparable, as ROC curves were not significantly different (*p* > 0.05).

### 3.3. Performance of the DMS for Ruling-Out NSTEMI

In the group of patients assessed by using the 0h/1h ESC algorithm (group 1), rule-out was achieved in 34 out of 51 patients (66.7%), among whom two false-negative cases were identified. Immediate rule-out (hs-cTnT < LoD) was feasible in 13 patients (25.4%), whereas it was not applicable in six patients (11.7%) due to early presentation (<3 h from chest pain onset). On the other hand, the application of the DMS correctly identified (ruled in) all NSTEMI cases. Moreover, the DMS enabled immediate rule-out in 29 out of 51 patients (56.9%) and in 32 out of 51 patients (62.7%) when using copeptin cut-offs of <10 and <14 pmol/L, respectively.

**Figure 6:**
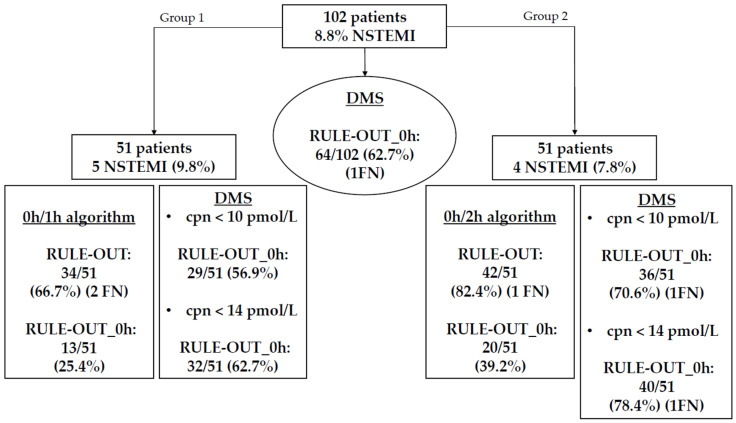
Schematic representation of the diagnostic efficacy of the Dual Marker Strategy (DMS) for the rule-out of non-ST elevation myocardial infarction (NSTEMI) in the overall population (copeptin < 10 pmol/L and hs-cTnT < 14 ng/L) and in groups 1 and 2 compared to the hs-cTnT 0h/1h and 0h/2h algorithms. Rule-out_0h denotes patients ruled out upon presentation (time 0 h). cpn = copeptin; DMS = Dual Marker Strategy; FN = false negative(s); NSTEMI = non-ST elevation myocardial infarction.

Similarly, in the group of patients assessed by using the 0h/2h ESC algorithm (group 2), rule-out was accomplished in 42 out of 51 patients (82.3%), with one case subsequently identified as false negative. Immediate rule-out was feasible in 20 patients (39.2%), while the algorithm could not be applied in one subject (2%) owing to early presentation. On the contrary, application of the DMS allowed immediate rule-out in 36 out of 51 patients (70.6%) when a copeptin cut-off of <10 pmol/L was applied and in 40 out of 51 patients (78.4%) when using a copeptin cut-off of <14 pmol/L. At each of these copeptin cut-offs, one false-negative case was observed (Figure 6).

## 4. Discussion

In the present prospective, randomized diagnostic accuracy study, we have shown that in patients presenting to the ED with chest pain, measurement of copeptin increased diagnostic performance of hs-cTnT as a single biomarker. Moreover, a DMS combining measurement of copeptin and hs-cTnT upon presentation demonstrated comparable diagnostic performance to the ESC-recommended 0h/1h and 0h/2h algorithms for the early rule-out of NSTEMI.

Currently recommended ESC hs-cTn algorithms provide high diagnostic accuracy for the diagnosis of NSTEMI; however, the requirement for a second measurement at 1 or 2h may incur delays in establishing a definitive decision. Given that the diagnostic performance of the DMS, combining copeptin with hs-cTnT upon patient presentation, appears comparable to that of standard algorithms, in terms of sensitivity and NPV, its application may allow immediate rule-out of NSTEMI in a greater proportion of patients. This is of particular importance in the ED, that not only manages a high volume of undifferentiated patients but also represents the first point of medical contact for patients with chest pain and bears responsibility for their early disposition to the appropriate level of care [22]. Early identification of low-risk patients among individuals presenting with potentially life-threatening conditions may decrease adverse outcomes associated with delayed management of high-risk patients, while also reducing overcrowding [23]. In such an intensive environment, diagnostic challenges demand easily applicable stratification tools aiming to expedite clinical assessment, improve patient outcomes and increase patient satisfaction [24,25]. Early rule-out of low-risk patients or of patients with other mimickers of ACS is equally important with early rule-in of high-risk patients so as to alleviate overcrowding, prevent unnecessary examinations and delays in patient assessment and provide appropriate management [26].

In our study, we examined the added value of copeptin on the diagnostic performance of hs-cTnT upon patient presentation, applying three different copeptin cut-off levels (<10, <14 and <20 pmol/L), and demonstrated that the addition of copeptin increased sensitivity and NPV of hs-cTnT as a single biomarker. Additionally, we evaluated the performance of the DMS and demonstrated that thresholds of 10 and 14 pmol/L yielded identical sensitivity (88.9%) and similar NPV (≈98.5%) for the rule-out of NSTEMI. On the contrary, when a higher cut-off (20 pmol/L) was applied, specificity and PPV increased, albeit with a negative impact on NPV (96%). These findings are in line with the 2015 ESC guidelines for the management of ACS in patients presenting without persistent ST-segment elevation, which recommend a copeptin cut-off of <10 pmol/L in combination with conventional troponin in order to enhance sensitivity and NPV for the early rule-out of NSTEMI [11].

Another significant finding of our study is that the DMS using a copeptin cut-off of <10 pmol/L and a hs-cTnT cut-off of 14 ng/L exhibited higher NPV compared to the 0h/1h and 0h/2h hs-cTnT algorithms. In fact, the application of DMS accurately ruled out a 2.23-fold and a 1.8-fold greater proportion of patients compared to the 0h/1h and the 0h/2h algorithm, respectively. More importantly, this was achieved upon ED presentation, without the need for conducting serial measurements. There have been previous reports in the literature about direct comparisons of the DMS with the 0h/1h [21,27,28] and 0h/2h algorithms [28,29]; yet, the reported results are somewhat conflicting. Our findings are in line with earlier studies performed either with hs-cTnT [21] or hs-cTnI [27,29], supporting the application of a DMS as an alternative early rule-out strategy in patients with suspected AMI. In contrast, Wildi et al. reported lower diagnostic performance of the DMS [28]; however, that was a retrospective study that included patients with STEMI. On the other hand, our study included unselected patients with chest pain, excluding those with STEMI, thereby reflecting a rather more appropriate clinical setting for the application of a DMS. Although the latest ESC guidelines on the management of patients with ACS do not favor the use of copeptin when hs-cTn assays are available [30], a recent critical appraisal called for further investigation into its additive diagnostic role, as the DMS may optimize patient management and disposition in the ED setting [31].

In the present study, we did not compare the diagnostic performance of the DMS with that of a single-marker strategy based on a single measurement of hs-cTn upon presentation (cut-off < lower limit of detection). However, previous studies have suggested higher [32,33] and equally safe rule-out rates for NSTEMI with the DMS compared to a single baseline hs-cTn measurement [34,35], highlighting the potential of the DMS to facilitate immediate rule-out of NSTEMI in a greater proportion of patients [35] or in early presenters [33].

In our study, measurement of copeptin on top of hs-cTnT upon presentation did not increase overall diagnostic performance, as indicated by the AUC, compared to the measurement of hs-cTnT alone. This aligns with our findings on the diagnostic performance analysis of hs-cTnT alone and in combination with copeptin upon presentation, where adding copeptin to hs-cTnT did not increase specificity and PPV of hs-cTnT as a single biomarker. This implies that a DMS may serve as a useful algorithm for the early rule-out of patients with suspected ACS, while rule-in requires further diagnostic investigations. Prior studies, which evaluated the diagnostic utility of copeptin in combination with hs-cTn at presentation using ROC analysis, have yielded contradictory results. Interestingly, some investigators have reported an improvement in AUC with the addition of copeptin to hs-cTn for the diagnosis of NSTEMI, both in patients presenting with suggestive symptoms irrespective of time from symptom onset [29,36,37] and in early presenters [38]. Nonetheless, this finding has not been consistently reproduced [21,33,39,40]. It should be noted that ROC analysis offers an overall view of the discriminatory performance of a biomarker for the diagnosis of a pathological condition by taking both sensitivity and specificity into account. However, when evaluating the diagnostic efficacy of an algorithm for the exclusion of NSTEMI, statistical metrics such as sensitivity and NPV may more accurately reflect the clinical utility of a rule-out strategy. Accordingly, although copeptin did not provide any additional diagnostic value for the detection of NSTEMI in terms of overall diagnostic performance, it could still serve as a supplementary “grouping” biomarker incorporated in the context of a DMS for the immediate rule-out of low-risk patients with a low pre-test probability of NSTEMI.

Although high-sensitivity troponin protocols offer undisputable benefits in the diagnostic workup of patients with suspected ACS, ongoing research focuses on their improvement, aiming to minimize the time interval from first medical contact to definite management [41,42]. Our findings suggest that incorporating copeptin into the diagnostic evaluation of patients with chest pain may facilitate risk-stratification and decision-making processes and enable instant and safe rule-out of low-risk patients. Special populations likely to benefit further from the application of a DMS are early presenters, patients with chronic kidney or coronary artery disease and patients with mild elevation of cardiac troponins. Beyond the clinical benefits, its use may reduce healthcare costs and hospital resources by preventing unnecessary examinations. In addition, the establishment of a DMS may also reduce length of stay and overcrowding in the ED.

Our study has several limitations. First, it is a single-center study that included a relatively small sample size, which may have affected the strength of our results, as indicated by wide confidence intervals and low sensitivities. Furthermore, our population consisted of predominantly low-risk individuals [median Heart Score 4 (2–5)], and the incidence of NSTEMI was low (8.8%) compared to that reported in other studies [32]. Hence, the generalizability of the data presented in our study is limited and warrants further investigation and validation in larger-scale multicenter trials.

## 5. Conclusions

In conclusion, we have demonstrated that, in patients presenting to the ED with symptoms suggestive of AMI, the application of a DMS that combines copeptin and hs-cTnT upon ED arrival could effectively aid in the early rule-out of NSTEMI. Since the diagnostic performance of the combined strategy appears to be comparable to that of the guideline-recommended hs-cTnT algorithms, the incorporation of copeptin into the initial diagnostic workup in combination with hs-cTnT may be a reasonable supplementary approach. Nevertheless, larger, well-designed, multi-center studies are required in order to provide robust evidence regarding the diagnostic utility of a copeptin-based DMS algorithm for NSTEMI.

## Figures and Tables

**Table 1:** Baseline characteristics.

	Total Cohort (n = 102)	NSTEMI (n = 9)	No NSTEMI (n = 93)	*p* Value
**Demographics**				
Age, years (Mean ± SD)	57.7 ± 18.4	61 ± 13.2	57.3 ± 18.8	0.628
Male sex, n (%)	61 (59.8)	5 (55.6)	56 (60.2)	0.785
**Clinical characteristics, Median (IQR)**				
SBP, mmHg	145 (134–157)	150 (134–170)	145 (134–156)	0.429
DBP, mmHg	82 (73–90)	88 (73–90)	82 (74–90)	0.479
HR, bpm	80 (73–90)	80 (74–90)	80 (73–90)	0.925
SpO2, %	97 (96–98)	97 (95–98)	97 (96–98)	0.481
EF, %	60 (50–60)	55 (50–60)	60 (50–60)	0.417
**Laboratory variables, Median (IQR)**				
hs-cTnT, ng/L	6.40 (4–12.5)	32.3 (7.0–82.0)	6.30 (4.0–11.1)	**0.020**
Copeptin, pmol/L	7.16 (4.50–12.11)	18.11 (14.62–22.41)	6.99 (4.5–10.1)	**0.046**
**ECG, n (%)**				
Normal	62 (60.8)	1 (11.1)	61 (65.6)	**0.011**
ST depression	5 (4.9)	2 (22.2)	3 (3.3)	**0.012**
T negative	7 (6.9)	2 (22.2)	5 (5.4)	**0.022**
ST non-specific abnormalities	11 (10.8)	3 (33.3)	8 (8.6)	0.056
**Comorbidities, n (%)**				
Smoking	45 (44.1)	5 (55.6)	40 (43)	0.592
Family history of cardiac disease	23 (22.5)	3 (33.3)	20 (21.5)	0.418
Coronary artery disease	25 (24.5)	4 (44.4)	21 (22.6)	0.145
Hypertension	40 (39.2)	4 (44.4)	36 38.7)	0.737
Hyperlipidemia	43 (42.2)	7 (77.8)	36 (38.7)	**0.023**
Diabetes mellitus	27 (26.5)	4 (44.4)	23 (24.7)	0.201
Heart failure	11 (10.8)	2 (22.2)	9 (9.7)	0.247
COPD	11 (10.8)	2 (22.2)	9 (9.7)	0.247
Stroke	3 (2.9)	2 (22.2)	1 (1.1)	**<0.001**
**Medication, n (%)**				
BBs	34 (33.3)	5 (55.6)	29 (31.2)	0.139
ARBs	36 (35.3)	5 (55.6)	31 (33.3)	0.183
ACE inhibitors	6 (5.9)	0 (0)	6 (6.4)	0.432
Antiplatelets	33 (32.4)	5 (55.6)	28 (30.1)	0.119
Statins	41 (41.4)	7 (77.8)	34 (36.5)	**0.016**
**Time onset of chest pain, n (%)**				**<0.001**
<1 h	7 (6.9)	3 (33.3)	4 (4.3)	
1–2 h	7 (6.9)	2 (22.2)	5 (5.4)	
2–3 h	11 (10.8)	2 (22.2)	9 (9.7)	
>3 h	77 (75.5)	2 (22.2)	75 (80.6)	
**Duration of chest pain, n (%)**				0.944
<10 min	15 (14.7)	1 (11.1)	14 (15)	
10–30 min	12 (11.8)	1 (11.1)	11 (11.8)	
>30 min	75 (73.5)	7 (77.8)	68 (73.1)	
**Heart score, Median (IQR)**	4 (2–5)	7 (6–7)	3 (2–4)	**<0.001**
1 (0–3), n (%)	50 (49)	0	50 (53.8)	
2 (4–6), n (%)	43 (42.2)	3 (33.3)	40 (43)	
3 (≥7), n (%)	9 (8.8)	6 (66.7)	3 (3.2)	
**Outcome**				
Admission, n (%)	31 (30.4)	9 (100)	22 (23.6)	**<0.001**

ACE = angiotensin-converting enzyme; ARBs = angiotensin II receptor blockers; BBs = beta blockers; COPD = chronic obstructive pulmonary disease; bpm = beats per minute; DBP = diastolic blood pressure; ECG = electrocardiogram; EF = ejection fraction; HR = heart rate; hs-cTnT = high-sensitivity cardiac troponin T; IQR = interquartile range; n = number; NSTEMI = non-ST elevation myocardial infarction; SBP = systolic blood pressure; SD = standard deviation; SpO2 = peripheral oxygen saturation.

**Table 2:** Diagnostic performance of hs-cTnT (<14 ng/L) and copeptin as single biomarkers and of the Dual Marker Strategy (DMS) for the diagnosis of non-ST-elevation myocardial infarction (NSTEMI) upon patient presentation when applying different cut-off values for copeptin (cpn < 10 pmol/L, <14 pmol/L and <20 pmol/L) in combination with high-sensitivity cardiac troponin T (hs-cTnT) < 14 ng/L in the overall population. hs-cTnT_0h and cpn_0h denote biomarker levels upon patient presentation (time 0). Values are expressed as percentages with corresponding 95% confidence intervals.

Study Population	Copeptin Cut-Off	Sensitivity	NPV	Specificity	PPV	Accuracy
hs-cTnT_0h	hs-cTnT < 14	66.7	96.3	83.9	28.5	82.4
		29.9–92.5	91.2–98.5	74.8–90.7	17.2–43.4	73.6–89.2
cpn_0h	(cpn < 10)	77.8	97.2	75.3	23.3	75.5
		40–97.2	91.1–99.2	65.2–83.6	15.6–33.3	66–83.5
cpn_0h	(cpn < 14)	77.8	97.5	83.9	31.7	83.3
		40–97.2	92–99.2	74.8–90.7	20.7–45.4	74.7–90
cpn_0h	(cpn < 20)	33.4	93.2	88.2	21.4	83.3
		7.5–70	89.6–95.6	79.8–94	8.5–44.4	74.7–90
hs-cTnT_0h_cpn_0h	(cpn < 10)	88.9	98.5	68.8	21.6	70.6
		51.75–99.72	90.94–99.76	58.37–78.02	15.87–28.75	60.75–79.2
hs-cTnT_0h_cpn_0h	(cpn < 14)	88.9	98.6	76.3	26.7	77.4
		51.75–99.72	91.77–99.78	66.4–84.5	19.1–35.9	68.11–85.14
hs-cTnT_0h_cpn_0h	(cpn < 20)	66.7	96	78.5	23.1	77.4
		29.93–92.51	90.57–98.40	68.76–86.34	14.09–35.42	68.11–85.14

cpn = copeptin; hs-cTnT = high-sensitivity cardiac troponin T; NPV = negative predictive value; PPV = positive predictive value.

**Table 3:** Diagnostic performance of the Dual Marker Strategy (DMS) for the diagnosis of non-ST elevation myocardial infarction (NSTEMI) upon patient presentation when applying different cut-off values for copeptin (cpn < 10 pmol/L, <14 pmol/L and <20 pmol/L) in combination with high-sensitivity cardiac troponin T (hs-cTnT) < 14 ng/L in patients allocated to the 0h/1h algorithm (group 1). Results are presented alongside those of the hs-cTnT 0h/1h algorithm. hs-cTnT_0h and cpn_0h denote biomarker levels upon patient presentation (time 0). Values are expressed as percentages with corresponding 95% confidence intervals.

Group 1	Copeptin Cut-Off	Sensitivity	NPV	Specificity	PPV	Accuracy
hs-cTnT_0h/1h		60	95.6	95.6	52.6	90.8
		14.66–94.73	88.06–98.45	85.16–99.47	19.47–83.57	77.10–97.66
hs-cTnT_0h_cpn_0h	(cpn < 10)	100	100	63	22.7	66.67
		47.82–100	88.06–100	47.55–76.79	16.78–30.02	52.08–79.24
hs-cTnT_0h_cpn_0h	(cpn < 14)	100	100	69.6	26.3	72.55
		47.82–100	89.11–100	54.25–82.26	18.75–35.60	58.26–84.11
hs-cTnT_0h_cpn_0h	(cpn < 20)	60	94.3	71.7	18.8	70.59
		14.66–94.73	84.74–98	56.54–84.01	8.97–35.08	56.17–82.51

cpn = copeptin; hs-cTnT = high-sensitivity cardiac troponin T; NPV = negative predictive value; PPV = positive predictive value.

**Table 4:** Diagnostic performance of the Dual Marker Strategy (DMS) for the diagnosis of non-ST elevation myocardial infarction (NSTEMI) upon patient presentation when applying different cut-off values for copeptin (cpn < 10 pmol/L, <14 pmol/L and <20 pmol/L) in combination with high-sensitivity cardiac troponin T (hs-cTnT) < 14 ng/L in patients allocated to the 0h/2h algorithm (group 2). Results are presented alongside those of the hs-cTnT 0h/2h algorithm. hs-cTnT_0h and cpn_0h denote biomarker levels upon patient presentation (time 0). Values are expressed as percentages with corresponding 95% confidence intervals.

Group 2	Copeptin Cut-Off	Sensitivity	NPV	Specificity	PPV	Accuracy
hs-cTnT_0h/2h		75	95.8	97.9	64	96.09
		19.41–99.37	85.22–98.93	88.71–99.95	14.10–95.05	86.56–99.52
hs-cTnT_0h_cpn_0h	(cpn < 10)	75	97.2	74.5	20	74.5
		19.41–99.37	86.41–99.48	59.65–86.06	10.59–34.55	60.37–85.67
hs-cTnT_0h_cpn_0h	(cpn < 14)	75	97.5	83	27.3	82.3
		19.41–99.37	87.67–99.53	69.19–92.35	13.84–46.68	69.13–91.6
hs-cTnT_0h_cpn_0h	(cpn < 20)	75	97.6	85.1	30	84.3
		19.41–99.37	87.95–99.55	71.69–93.80	15–51	71.41–92.98

cpn = copeptin; hs-cTnT = high-sensitivity cardiac troponin T; NPV = negative predictive value; PPV = positive predictive value.

## Data Availability

The data are available from the corresponding author upon reasonable request.

## Abbreviations

The following abbreviations are used in this manuscript:

ACS	Acute Coronary Syndrome
AMI	Acute Myocardial Infarction
AUC	Area Under the Curve
AVP	Arginine Vasopressin
CI	Confidence Interval
DMS	Dual Marker Strategy
ECG	Electrocardiogram
ED	Emergency Department
EDTA	Ethylenediaminetetraacetic Acid
ESC	European Society of Cardiology
hs-cTn	High-sensitivity Cardiac Troponin
hs-cTnT	High-sensitivity Cardiac Troponin T
IQR	Interquartile Range
LoB	Limit of Blank
LoD	Limit of Detection
LoQ	Limit of Quantification
NPV	Negative Predictive Value
NSTEMI	Non-ST Elevation Myocardial Infarction
PPV	Positive Predictive Value
proAVP	Prohormone of Arginine Vasopressin
ROC	Receiver Operating Characteristic
SD	Standard Deviation
STARD	Standards for Reporting Diagnostic Accuracy
STEMI	ST Elevation Myocardial Infarction
TRACE	Time-Resolved Amplified Cryptate Emission
UA	Unstable Angina
URL	Upper Reference Limit
